# Supplementary material for: Recombinant Globular Domain of TcpA Pilin from Vibrio cholerae El Tor: Recovery from Inclusion Bodies and Structural Characterization
Source: Life (Basel). 2022 Nov 7;12(11):1802. doi: 10.3390/life12111802 (PMC9695179; doi:10.3390/life12111802)
Supplement: Supplementary file 1 [file life-12-01802-s001.zip › life-1909920-supplementary.pdf]

Supplementary Materials

leader sequence — α1-N helix

(a) TcpA^{ET}	MQLLKQLFKKKFVKEEHDKKTGQEGMTLLEVIIVLGIMGVVSAGVVTLAQRAIDSQNMTK	60
(b) TcpA^{ET}-C	-------------------------------MGSSHHHHHHSSGL-VPRGSHMDSQNMTK	28
(c) TcpA^{CIRS}-C	---MHHHHHHSQNMTK	13

α1-C helix

(a) TcpA^{ET}	AAQNLNSVQIAMTQTYRSLGNYPATANA**N**AATQLANGLVSLGKVSADEAKNPFTGTAMGI	120
(b) TcpA^{ET}-C	AAQNLNSVQIAMTQTYRSLGNYPATANA**N**AATQLANGLVSLGKVSADEAKNPFTGTAMGI	88
(c) TcpA^{CIRS}-C	AAQNLNSVQIAMTQTYRSLGNYPATANA**S**AATQLANGLVSLGKVSADEAKNPFTGTAMGI	73

(a) TcpA^{ET}	FSFPRNSAANKAFAITVGGLTQAQCKTLVTSVGDMFPFINVKEGAFAAVADLGDFETSVA	180
(b) TcpA^{ET}-C	FSFPRNSAANKAFAITVGGLTQAQCKTLVTSVGDMFPFINVKEGAFAAVADLGDFETSVA	148
(c) TcpA^{CIRS}-C	FSFPRNSAANKAFAITVGGLTQAQCKTLVTSVGDMFPFINVKEGAFAAVADLGDFETSVA	133

(a) TcpA^{ET}	DAATGAGVIKSIAPGSANLNLTNITHVEKLCTGTAPFTVAFGNS	224
(b) TcpA^{ET}-C	DAATGAGVIKSIAPGSANLNLTNITHVEKLCTGTAPFTVAFGNS	192
(c) TcpA^{CIRS}-C	DAATGAGVIKSIAPGSANLNLTNITHVEKLCTGTAPFTVAFGNS	177

Figure S1. The sequences of TcpA proteins (alignment by BLAST, https://www.uniprot.org/blast [6]; the leader sequence and the helixes α1-N and α1-C (parts of the single long α1-helix) in TcpA^{ET} are marked with black arrows; the amino acid residues identical in all three sequences are highlighted in grey; the substituted amino acid residue is typed in bold and marked with a red arrow:

(**a**) – typical TcpA El Tor (UniProt Q60153: from *Vibrio cholerae* serotype O1, strain ATCC 39315 / El Tor Inaba N16961);

(**b**) – TcpA^{ET}-C globular domain used for the crystal structure determination (PDB 3HRV: from *V. cholerae* serotype O1, strain C6706, biovar El Tor Inaba [16]);

(**c**) – TcpA^{CIRS}-C globular domain used in the current study (*V. cholerae* serotype O1, strain M1429, biovar El Tor [17]).

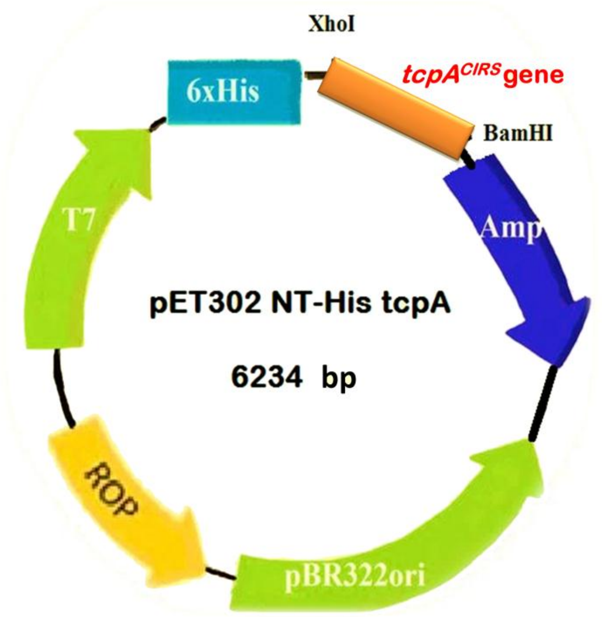

Figure S2. Schematic presentation of the expression plasmid pET302 NT-His tcpA^{CIRS} (adapted from [17]).

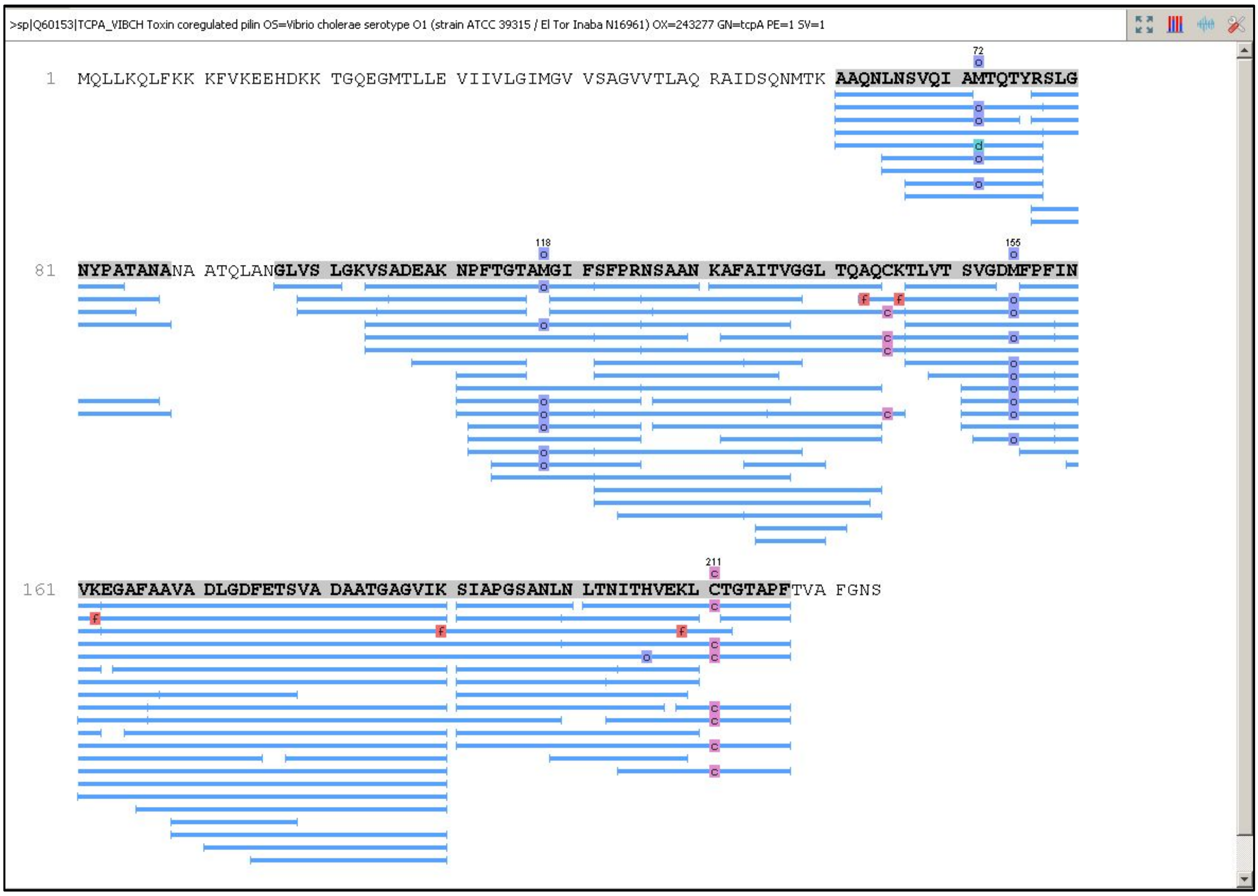

Figure S3. Mass spectrometry analysis of recombinant TcpA-C. The full protein sequence of the typical TcpA El Tor (UniProt Q60153: from *Vibrio cholerae* serotype O1, strain ATCC 39315 / El Tor Inaba N16961) is shown in the top line. The identified part of this sequence is shown in gray. The unique peptides of the recombinant protein TcpA-C identified by mass spectrometry are shown in blue under the sequence. The variable modifications of TcpA-C are indicated by letters in squares (o – oxidation of Met, d – dioxidation of Met, f – formylation, and c – carbamidomethylation of Cys).

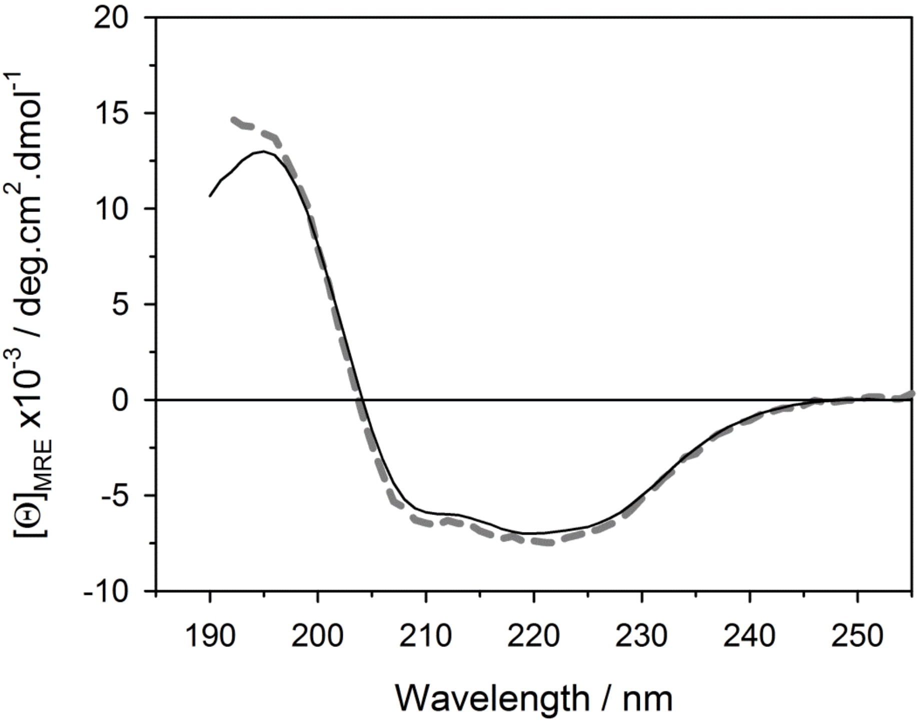

Figure S4. Far-UV CD spectra of TcpA-C purified and refolded from inclusion bodies (solid black line) and purified from the soluble fraction (dashed grey line), 20 mM Tris-HCl, pH 8.9, 150 mM NaCl.

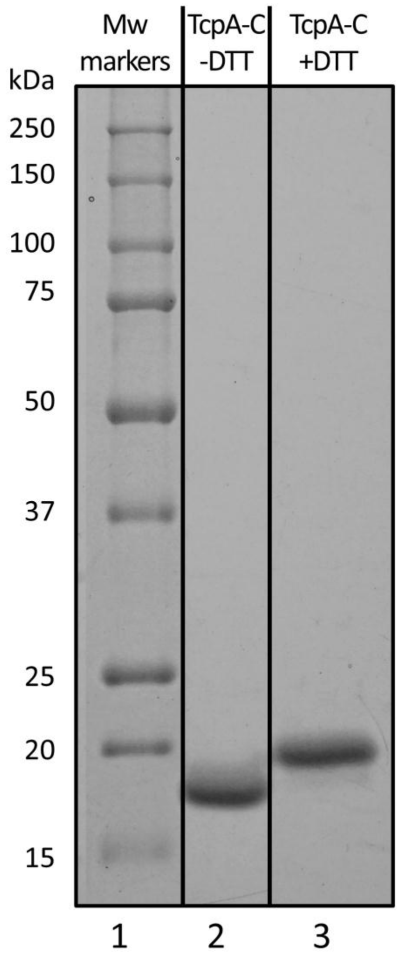

Figure S5. SDS-PAGE of TcpA-C purified from inclusion bodies: molecular weight markers (lane 1); TcpA-C in the standard buffer (20 mM Tris-HCl, pH 8.9, 150 mM NaCl) (lane 2); TcpA-C in the standard buffer supplemented with 30 mM DTT (lane 3).

Table S1. The TcpA-C secondary structure contents (%).

**Type of secondary structure**	**In solution (calculation from Far-UV CD spectrum of TcpA^{CIRS}-C)**			**In crystal (calculation from crystal structure (chain B) of TcpA^{ET}-C** [16]**)**		
	**BeStSel** [21]	**CDNN CD deconvolution software 2.1** [22]	**K2D3 web server** [23]	**BeStSel** [21]	**DSSP** [24]	**SELCON** [25]
α	20	24	1	27	33	33
β	28	22	44	19	19	19
other	52	54	55	54	47	47
